# Barriers and Facilitators to Technology Use Among Caregivers of Homebound Older Adults: Descriptive Qualitative Study

**DOI:** 10.2196/96226

**Published:** 2026-11-06

**Authors:** Carina Katigbak, Esha Shenoy, Padmavathy Ramaswamy, Jessica Lee, Kathrin Milbury, Katie Scott

**Affiliations:** 1 Department of Research, Cizik School of Nursing, The University of Texas Health Science Center at Houston, 6901 Bertner Ave, Houston, TX, 77007, United States; 2 Dell Medical School, The University of Texas at Austin, Austin, TX, United States, 1 7135002206; 3 Department of Graduate Studies, Cizik School of Nursing, The University of Texas Health Science Center at Houston, Houston, TX, United States; 4 Department of Internal Medicine, McGovern School of Medicine, The University of Texas Health Science Center at Houston, Houston, TX, United States; 5 Department of Behavioral Science, Division of Cancer Prevention and Population Sciences, The University of Texas MD Anderson Cancer Center, Houston, TX, United States; 6 CarePartners, Houston, TX, United States

**Keywords:** caregivers, health promotion, technology, qualitative, older adults

## Abstract

**Background:**

Homebound older adults (≥60 y) and their informal family caregivers face multiple challenges in meeting their instrumental, emotional, and informational needs, which result in lower functional status, multiple comorbidities, and poorer mental health. Caregivers are tasked with providing uncompensated care for medically complex older adults, often without formal health care training. This circumstance may translate to high levels of burden and greater vulnerability to social isolation and loneliness, which may precipitate chronic stress and compromise caregiver health. In recent years, numerous health-focused technologies assisting with health-promoting behaviors such as physical activity, stress management, and nutritional tracking have emerged. However, little is known about how these tools are adopted by family caregivers of homebound older adults.

**Objective:**

This descriptive qualitative study aimed to explore how caregivers of homebound older adults perceive technology in the context of supporting caregiving and aging in place through focus groups and one-on-one interviews.

**Methods:**

We conducted a descriptive qualitative study using in-person and online video conference focus group methodology with 40 individuals (32 caregivers, 6 clinicians, and 2 additional key informants) to explore perspectives on caregivers’ use of technology to support their caregiving activities. Participants completed a brief sociodemographic questionnaire prior to all interviews. Procedures for thematic analysis were used to analyze the data and identify emerging themes.

**Results:**

Three main themes (and related subthemes) emerged from the data: (1) these technologies are not designed for people like us, (2) balancing the scales, and (3) preserving a sense of self. Caregivers’ experiences with technology are marked by a paradox: the same technologies that can ease caregiving activities may also impose new burdens through additional costs, uncertainty, learning demands, and emotional strain.

**Conclusions:**

Caregivers should not navigate these technological tensions alone. Realizing the promise of technology requires more than innovative tools—it demands caregiver-centered design, accessible and equitable implementation strategies, and sustained clinical and health system support to ensure that technology alleviates, rather than amplifies, caregiver burden.

## Introduction

By 2040, one in five Americans (nearly 78.5 million people) will be aged 65 years or older, a 17% increase from 2022 [1]. While this growth may be attributed to medical advancements and longer life expectancy, a large majority of US older adults also live with multiple comorbidities and functional impairments [2] related to meeting needs for activities of daily living that require assistance from informal family caregivers [3]. Of this population, more than 7 million US older adults [4] are homebound, which is defined as individuals who never or rarely leave their homes because of their complex care needs [5]. Homebound individuals have a higher chronic disease burden along with reduced access to primary care (due to their inability to leave home). As such, informal family caregivers (herein referred to as caregivers) play a critical role in acting as the primary link between the health care system and homebound older adults requiring comprehensive assistance.

Caregivers often lack formal health care training but are nonetheless tasked with caring for medically complex older adults, a circumstance that may precipitate the harmful effects of chronic stress [6]. The general literature on caregivers of older adults identifies that they face challenges in meeting their tangible, emotional, and informational needs [7,8], which consequently increase their risk for diminishing functional status, multiple comorbidities, and poorer mental health [9]. High burden levels, greater vulnerability to social isolation, and loneliness [10] negatively impact their quality of life [11,12] and may potentially harm the interpersonal dynamic between caregiver and care recipient [13,14]. Similarly, some caregivers of older adults who are homebound shared that rewarding feelings of duty and gratitude in being able to care for their loved ones [15] are frequently overshadowed by providing round-the-clock care and its impact on their other interests and obligations [16]. Over the course of the caregiving journey, caregivers’ health can become severely compromised [17-19], underscoring the critical need to support and maintain caregiver health and well-being. Interventions and strategies for caregivers to maintain their own health largely focus on stress management techniques and psychoeducational interventions [20]. A systematic review of interventions targeting caregivers reported greater effectiveness in increasing caregiver knowledge and ability rather than mitigating caregiver burden and depression [21]. Although digital health tools are increasingly marketed to promote caregiver health, and these may be used as promising tools to improve caregiver outcomes, a recent review noted that of the over 200,000 health-focused mobile apps, only 44 were targeted toward caregivers [22]. Remote and digital tools may be especially useful for caregivers of homebound adults, as home-based medical care has proven to alleviate the burden of their fragmented care [15]. These digital apps largely focus on the process of caregiving in 3 categories of support: information and resources, family communication, and caregiver-recipient interactions. Few comprehensively addressed domains of support and seldom addressed specific stress reduction strategies for reducing caregiver burden [22]. Moreover, a scoping review determined that caregiver apps to support care planning and coordination for those with multiple chronic conditions are underdeveloped [23]. Digital solutions (using computer programs, smart-enabled devices, and the internet to improve efficiencies at home) are not limited to mobile apps alone but also extend to telehealth consultations [24], in addition to various devices that those in the caregiving community use for tracking/monitoring [23,25] purposes, such as GPS trackers, wearable technologies, and apps (eg, smart watches, Find My [Apple Inc], and AirTag [Apple Inc]).

Understanding patterns of technology use and device adoption across diverse caregiver populations is increasingly important for enhancing geriatric care and can guide recommendations about appropriate technology interventions [26]. While health-focused technologies exist and continue to evolve rapidly, these technologies must be acceptable, accessible, equitable, and responsive to caregivers’ needs [19,27]. Thus, our study sought to describe how family caregivers of homebound older adults use technology to support their caregiving activities, specifically identifying barriers and facilitators to its use; understanding these perspectives is crucial for developing user-centered interventions that optimize the use of technology to support caregivers and encourage high-quality care.

## Methods

### Study Design

We conducted a qualitative descriptive study to directly capture participants’ perspectives on how technology is used to support caregiving activities. Using a community-engaged research approach, we collaborated with Care Partners, a Houston-based community services and advocacy group that delivers high-quality programming for older adults and people with dementia, including family caregivers (such as support groups, educational programming, and in-home visits for caregiver consultation). The organization serves over 2500 older adults in the Houston area. We sought to examine caregivers’ experiences using technology to facilitate their caregiving activities and triangulated these data with information shared by clinicians and key informants—specifically around barriers and facilitators to integration and consistent use. The study followed guidelines for the COREQ (Consolidated Criteria for Reporting Qualitative Research).

### Participant Recruitment

From July to November 2023, a purposive sample of caregivers and key informants were recruited from CarePartners’ roster of volunteer caregivers, clinicians, and community key informants to triangulate data from multiple sources. Consistent with Braun and Clarke reflexive thematic analysis [28], sample adequacy was evaluated based on the richness of the data and the analytic objectives rather than the achievement of data saturation. Recruitment continued until the research team judged that the dataset provided sufficient depth and breadth to address the research question.

Eligibility criteria consisted of the following: informal, unpaid, caregivers of older adults (>60 y old), who were aged over 21 years old, fluent in English, residing in the same home as the older adult, and providing more than 12 hours of care per week; clinicians currently working with CarePartners; and community key informants (eg, community leaders and elder care advocates). Multiple modalities were used to recruit participants: (1) engaging in direct outreach to caregivers participating in online support groups, sharing information with support group moderators, and joining online meetings to provide details about the study; (2) disseminating information about the study through a weekly electronic CarePartners newsletter, which is shared with its membership; and (3) posting physical flyers in common spaces. Prospective participants contacted CarePartners staff to complete an electronic form so that a member of the research team could follow up and determine study eligibility. A trained research nurse assessed participants for study eligibility, obtained informed consent, and scheduled an interview date and time with the participant.

### Researcher Positionality

The study team comprised investigators with expertise in nursing, medicine, psychology, public health, and qualitative methods—all of whom are experienced in working with patients with chronic illness and their families. We acknowledge that our clinical and research backgrounds, as well as our commitment to improving caregiver support and health equity, informed the framing of the research questions and interpretation of the findings. To promote reflexivity, team members engaged in ongoing discussions throughout data collection and analysis to examine assumptions, challenge interpretations, and consider alternative explanations. Coding and thematic development were conducted collaboratively, with analytic decisions documented to enhance transparency and ensure that findings remained grounded in participants’ accounts.

### Data Collection

#### Interview Procedures and Participant Consent

Participants were invited to engage in in-person or virtual interviews (via Zoom); caregivers and clinicians participated in 60- to 90-minute focus group interviews (comprising 6‐8 people per group), while key informants had individual online interviews to glean their unique perspectives on the phenomenon. Prior to initiating data collection, we solicited our community partners’ feedback for improving the qualitative interview tool. Participants were informed of the confidential and voluntary nature of their participation in the study and were informed that they could choose to withdraw at any time.

#### Demographic Survey

Eligible participants first completed a demographic survey (to characterize the caregiver sample and integrate with qualitative interpretation) in REDCap (Vanderbilt University) via a shared email link. In addition to sociodemographic information, we collected data on caregivers’ self-reported health (reported as “good,” “very good,” and “fair”) and history of chronic illness. Caregiver burden was assessed with the 4-item Zarit Burden Interview (ZBI-4) [29], an abbreviated version of the original 22-item instrument to screen for caregiver burden. Each item is rated on a 5-point Likert scale ranging from 0 (never) to 4 (nearly always), yielding a total score from 0 to 16, with higher scores indicating greater perceived caregiver burden. Consistent with the original validation study, ZBI-4 scores were analyzed as a continuous measure. Perceived social support from family and friends was measured by the 10-item Lubben Social Network Scale (LSNS) [30] where responses are scored on a 6-point scale (0‐5) and summed for total scores of 0 to 50. Higher scores indicate larger, supportive networks and lower risk of social isolation. The 14-item senior technology acceptance model [31] assessed attitudinal beliefs, control beliefs, health conditions, and gerontechnology anxiety. Each item is rated on a 10-point scale ranging from 1 (strongly disagree) to 10 (strongly agree). All items are summed to generate a cumulative score ranging from 14 to 140, where higher scores indicate a greater acceptance toward using technology.

#### Qualitative Interviews

Interviews were conducted by CK and PR, both investigators with prior experience in leading qualitative studies and conducting qualitative interviews. Both interviewers had no prior relationship with the study participants. In-person interviews were conducted in the community collaborator’s private meeting rooms, and all interviews were recorded with 2 digital audio recorders to prevent data loss. Virtual interviews were conducted using a secure, HIPAA (Health Insurance Portability and Accountability Act)-compliant version of Zoom (education and health care version); audio and video were captured with Zoom’s “record” and transcript functions.

### Data Analysis

Quantitative survey data collected in REDCap were exported to SPSS (version 29; IBM Corp) for data management and analysis. Descriptive statistics were performed to characterize the study sample. Qualitative interview data collected in person were professionally transcribed and reviewed by members of the research team, while virtual interviews for which transcript data were automatically transcribed were similarly reviewed to verify transcript accuracy. All data (including digital audio and Zoom meeting recordings and raw interview transcripts) were stored on a secure, institutional drive. Finalized transcripts were deidentified and uploaded to ATLAS.ti (version 5.22.1; Lumivero, LLC) to assist with analysis. Data were analyzed using methods for thematic analysis [28] to assist with addressing the goals of our study. Two members of the research team with prior qualitative research experience first read and reread the entire deidentified, transcribed interview dataset to become familiar with the data. Then, each coder independently developed initial codes (semantic and underlying) to represent interpretive labels for the data that addressed the research question. The codebook contained a comprehensive list of codes with exemplar excerpts from the dataset. Coders met upon completion of the initial coding to discuss diverging assumptions, interpretations, and conceptual insights. Disagreements between coders were treated as opportunities to explore deeper, underlying patterns within the data. After all data were coded, an initial thematic map was developed by grouping together multiple codes sharing similar underlying concepts into higher-order themes and subthemes. The research team then met to review and refine the initial themes, discussing how accurately these represented the coded data (and the entire dataset) and whether to discard or merge themes as needed. Detailed descriptions of the final themes and subthemes were then finalized.

### Trustworthiness

To ensure qualitative rigor and trustworthiness as described by Lincoln and Guba [32], we maintained a detailed audit trail, practiced reflexive memoing, held team discussions throughout the analytic process, and used data source triangulation methods to cross-validate the findings.

### Ethical Considerations

Participants were offered a US $100 gift card for their time and contribution to the study. Ethics board approval for human subjects research was provided by the UTHealth Houston Institutional Review Board (number HSC-SN-22‐0575). A trained research nurse (and member of the study team) obtained participants’ informed consent using REDCap’s e-consent feature. All participants were advised of the voluntary nature of study participation, encouraged to ask questions, and informed that they could withdraw from participation at any time.

## Results

A total of 40 individuals participated in the study. The sample consisted of 32 caregivers, 6 clinicians, and 2 key informants to comprise a total of 5 focus group interviews and 2 individual interviews; of these, 4 focus groups were held with caregivers (8 individuals per group), 1 with clinicians, while individual interviews were conducted with key informants.

### Demographic Survey

Demographic characteristics for caregiver participants are summarized in Table 1. Caregivers were an average age of 67.6 (SD 10.79) years, female (28/32, 87.5%), a combined category of Black Hispanic (14/32, 43.75%), college degree holders (12/32, 37.5%), and most (20/32, 62.5%) had an average annual income of US $30,000 to US $49,999. Data regarding care recipients were not collected. Caregivers had spent a mean of 7.15 (SD 3.31) years caring for their loved one. They reported having insufficient social connections according to the LSNS-Revised scale (mean 14.38, SD 4.54), while caregiver burden as measured through the Zarit Burden Interview was moderate (mean 7.44, SD 2.90). Finally, technology acceptance measured by the technology acceptance model questionnaire was relatively high (mean 95.00, SD 13.31) among participants, and most respondents (14/32, 43.75%) rated their health as “good.”

**Table 1.** Caregiver participant characteristics (n=32).

Characteristics	Value
Age (y), mean (SD)	67.63 (10.79)
Social engagement (LSNS^a^), mean (SD)	14.38 (4.54)
Caregiver burden (ZBI-4^b^), mean (SD)	7.44 (2.90)
STAM^c^, mean (SD)	95.00 (13.31)
Sex, n (%)	
Male	4 (12.5)
Female	28 (87.5)
Race/ethnicity, n (%)	
White	6 (18.75)
Non-Hispanic Black	6 (18.75)
Hispanic Black	14 (43.75)
Asian	6 (18.75)
Education, n (%)	
High school	2 (6.25)
Some college	2 (6.25)
College degree	12 (37.50)
Graduate degree or higher	6 (18.75)
Prefer not to answer	10 (31.25)
Income (US $), n (%)	
≤30,000	4 (12.5)
30,000-49,999	20 (62.5)
50,000-74,999	2 (6.25)
75,000 and more	4 (12.5)
Prefer not to answer	2 (6.25)
Self-rated health, n (%)	
Very good	16 (50)
Good	14 (43.75)
Fair	2 (6.25)
History of chronic illness, n (%)	
Diabetes	14 (43.75)
Hypertension	8 (25)
Overweight	10 (31.25)^c^

a LSNS: Lubben Social Network Scale*.*

b ZBI-4: 4-item Zarit Burden Interview.

c STAM: senior technology acceptance model*.*

### Qualitative Interviews

Through our qualitative interviews, participants shared an overarching sentiment that using technology to support their caregiving activities is a balancing act that is largely dictated by their care recipient’s needs. Overall, caregivers described a paradoxical relationship with technology, valuing its ability to provide practical assistance, enable emotional connection with others, and provide reassurance, while also recognizing that poorly designed, expensive, or difficult-to-learn technologies could increase the caregiving burden. As such, 3 themes emerged from the data illustrating the competing tensions regarding technology that exist for caregivers of homebound older adults: (1) These Technologies Aren’t Designed for People Like Us, Facing These Problems, (2) Balancing the Scales, and (3) Preserving a Sense of Self. The 8 related subthemes are further described below.

### Theme 1: These Technologies Aren’t Designed for People Like Us, Facing These Problems

#### Challenges to Technology Use Among Caregivers

Caregivers shared that they are faced with a technological conundrum; wherein most available technologies are not uniquely tailored to the caregiving experience, leaving caregivers to “pick and choose” what works for them at any given moment, while acknowledging that the investment of time and money to learn these technologies is a barrier to technology use. Three subthemes further illustrate this theme: (1) Cascading Costs of Technology Use, (2) Poor Technological Dexterity, and (3) Technology Unlocks a Black Box of Fears. These subthemes reflect a range of challenges related to accessing technology, required skillsets and abilities, and psychological barriers that are discussed in greater detail below.

#### Subtheme 1: Cascading Costs of Technology Use

Participants expressed a strong sentiment that using technology bears significant costs. These costs were not only limited to finances but also broadly included time and “mental bandwidth.” Caregivers highlighted monetary costs, both up-front and long-term, that hinder technology use. Immediate costs associated with purchasing equipment weighed heavily on participants’ decisions to commit to buying and using technology. “I’m willing to spend this much money on this thing and what if I don’t [use it]?” (P15; 79-year-old female participant). Although many articulated the direct benefits that certain technologies may contribute to enriching their daily activities, these appeared to be overshadowed by concerns about the time needed to identify suitable, cost-effective technology, the learning curve associated with becoming comfortable with the devices, and setting them up. For instance, a 78-year-old female participant (P9) noted the following:

*I’m afraid to try something new. And I don’t like to talk to the tech people about it, because I don’t even know what words to use or what to say. I don’t know what [device or app] to pick or where to start.*

Moreover, the belief that the time spent researching, acquiring, and learning how to use a new technology outweighs its potential benefit was shared. One 80-year-old male caregiver (P18) noted the following:

*…it’s just gonna be something new, and it’ll be there, and then pretty soon it’ll be old and just getting dust on it…. And you don’t want to spend it just spinning your wheels and not going any place.*

Insightfully, one 59-year-old male participant (P8) noted that devices lacking system interoperability (Apple iOS and Microsoft, for example) forced him to spend time trying to “make devices (and software) work together,” leading to frustration and disappointment with the technology that is marketed to simplify an aspect of one’s life. He further stated, “sometimes the technology itself is the lynchpin. It’s just it. It gets you on a glitch, and it gets you frustrated.” Compounded, these challenges serve as hindrances to technology use despite their potential benefits.

#### Subtheme 2: Poor Technological Dexterity

Poor technological dexterity, which encompasses challenges in quickly learning, adapting to, and effectively using technology, emerged as a second subtheme. Many caregivers shared that challenges with adopting and adapting to technology were related to lacking a baseline expertise and comfort with technology. These tasks were described as cumbersome, and many caregivers believed they were poorly equipped to engage in these activities. For instance, one 66-year-old female caregiver (P30) stated the following:

*I’m not always ready for the newest thing, you know, and I know most older adults aren’t ready for the newest thing, because it’s something else that they have to think about, you know, or they have the process, or it’s not that easy, you know, but you think differently when you tend to care for older adults*

Some caregivers stated that they lacked a degree of tech savviness, highlighting a generational digital divide as adult children or grandchildren were often consulted to address these gaps, as one 79-year-old female participant (P6) recounted that she relies on others stating she has “a son-in-law that he’s very technology savvy and so…he’s the one that put all of these things for me (laughs).” Specific activities that posed challenges included the following: managing passwords for multiple accounts, learning to use new devices, applications, and related platforms. Finally, physical impediments such as issues with eyesight and diminished manual dexterity were noted as significant detractors, with some caregivers emphasizing that these technologies are not designed with these age-related issues in mind. Many participants reported overcoming a steep learning curve to integrate technology into their lives; however, a 59-year-old male participant (P8) suggested that technology should dynamically adjust to its user’s unique needs:

*You look at the font because the older you get; our eyes get worse… I think technology needs to be set up in a way that one of your login things should be like, what’s your age? And then the font can adapt itself there. There should be an easy technology to be able to do that.*

For some caregivers, technology emphasized the digital divide, while others saw potential on how it may be used to meet aging adults where they are.

#### Subtheme 3: Technology Unlocks a Black Box of Fears

The final subtheme centers on a variety of technology-related fears. These fears range from uncertainties that are anticipatory in nature, such as feeling overwhelmed by the prospect of learning something new and viewing that as a stressful activity, to fears of breaking devices and their replacement costs.

*I’ve always been afraid of all these devices-I still am. I’m afraid of them. I don’t know what I think. I think I’m gonna touch them and they’re gonna break, and it’s got to cost me more money*

[P19; 71-year-old female participant].

Many respondents reported that despite the potential benefits of technology in their caregiving activities, they were nonetheless doubtful of their own abilities to learn new skills and invest time and energy. A 79-year-old female participant (P16) stated the following:

*...I think I’m pretty technologically savvy, but I don’t want to go down that route, because. I see it as right now as counterproductive timewise and energy wise. It’s frustrating to try and deal with it.*

Furthermore, a clinician (P11; 37-year-old female participant) noted that she often encounters caregivers who express the following fear:

*fear of perhaps messing something up, the fear of the unknown, you know,* “*maybe I don’t have the knowledge to be able to succeed with something that’s very technology based”*

In an effort to avert failure, these caregivers will choose to avoid technology altogether.

### Theme 2: Balancing the Scales

#### Facilitators of Technology Use in Caregiving

The theme “Balancing the Scales” illustrates caregivers’, clinicians’, and key informants’ perspectives that technology may serve to temper the complexities of their caregiving experiences. One 66-year-old female caregiver (P20) evoked dramatic imagery in describing their caregiving role as a juggling act where she is “juggling your [care recipient’s] life, our life, and my life.” The notion of holding multiple balls in the air was a sentiment shared by many caregivers, and technology was described across interviews as a tool to help alleviate the burdens associated with this juggling act. Participants shared their standpoints on factors that facilitate the use of technology to support aging in place, as represented through 3 subthemes described in further detail below: (1) Tailored Teaching Engages and Empowers Caregivers, (2) Technology Streamlines and Organizes Complex Daily Routines, and (3) Promoting Sense of Security.

#### Subtheme 1: Tailored Teaching Engages and Empowers Caregivers

Participants expressed the desire for tailored teaching to meet their needs. While most caregivers were not opposed to technology, some were hesitant as exemplified by the following quote:

*I’d be willing to look at them [devices] if someone trained me on how to use them; but to just jump into it without understanding the nuances...I’m not comfortable*

[P24; 80-year-old male participant].

Respondents cited varying modes of instruction they perceived as helpful. One 59-year-old male participant (P8) noted the importance of providing information that is relatable and salient for the end user:

*They [organization] send[s] out a newsletter, and it’s for seniors by seniors, and they do a lot of free technology training for seniors... make sure that you have someone providing that [information] within that person’s age range, because it makes the communication easier.*

Suggested strategies for tailored teaching included involving adult children and grandchildren (with whom participants would eventually interact using these devices) as trainers and hosting community events for individuals/caregivers with novice technological abilities. Further, one clinician (P12; female who did not provide her age) suggested that a stepwise teaching approach branching from a basic introduction to smartphones (and similar devices) to guided hands-on training to using various applications would be optimal for caregivers. Thus, multiple training modalities are necessary toward addressing caregivers’ needs for technological education and training.

#### Subtheme 2: Technology Streamlines and Organizes Complex Daily Routines

Caregivers described complex care routines alongside managing a functional household as activities that occupied their days. For many, the COVID-19 pandemic created a need to become technologically literate, as providers moved to telehealth modes of care delivery. This necessary change spurred some caregivers to become more receptive to an array of technological tools such as calendars to organize appointments, logs to document vitals and medication, patient care portals, online grocery delivery, and other online purchasing; these served to aid in streamlining care and organizing tasks of daily living. Telehealth visits were embraced by this group as some care recipients had mobility impairments. A 78-year-old female participant (P9) stated, “when I called to make the appointment with the doctor, I said, Yeah, give me the telehealth because I don’t know that I can get him out.” The convenience of telehealth was praised by many caregivers; however, some expressed frustration that providers no longer provided telehealth options or that insurance/Medicaid would no longer cover telehealth visits after the COVID-19 pandemic restrictions ended.

Many participants noted that their loved ones required round-the-clock supervision or care, making leaving their homes for simple errands a difficult task; therefore, grocery delivery services and online purchasing options greatly alleviated this burden. One participant remarked, “I don’t have to go to the grocery store now. I can have groceries delivered, and how easy you know, and convenient, you know that is” (P10; 52-year-old female participant). Another further reinforced the ease of online purchasing, emphasizing that it’s “so much more convenient, and it comes right away” (P8; 59-year-old male participant).

#### Subtheme 3: Promoting Sense of Security

With care recipients’ safety at the forefront of all participants’ perspectives, technology was frequently mentioned as a means of maintaining caregivers’ peace of mind related to their loved one’s safety and security. Many used cellphone-connected video monitoring systems to keep an eye on care recipients. A unique aspect of this technology is that it allows for care coordination between the caregiver and other family members. One caregiver described leaving her husband (care recipient) at her daughter’s home, where she could view a live camera-feed and easily check in on him,

*...so sometimes I go out and I say [to my daughter], " I don’t see him in the camera,” would you call? She can call because … it’s [video camera] connected to her house.*

[P6; 79-year-old female participant].

Others described repurposing technology like AirTags as wearable watches to create inconspicuous trackers to monitor care-recipients with memory loss without infringing on their sense of autonomy and independence. Many caregivers remarked that monitoring care-recipients, especially among those with memory issues and wandering tendencies, maintains safety while avoiding institutional placement. However, despite these generally favorable views toward remote monitoring to promote safety and autonomy, one caregiver expressed a strong belief that “surveilling someone is a denigrating violation of their integrity as a person to be tracking them around” (P5; 71-year-old female participant).

### Theme 3: Preserving a Sense of Self

Caregivers described using technology as a means of *Preserving a Sense of Self* amidst their all-consuming caregiving role. Participants shared that they personally used technology to buoy their sense of self through the following 2 subthemes of (1) Maintaining Social Connectedness and (2) Promoting Wellness/Self-Care, enabling them to persevere in their respective caregiving journeys.

#### Subtheme 1: Maintaining Social Connectedness

Caregivers reported using technology as a strategy to maintain social connections, though they conceded that technological connections are not a replacement for real-life connection. To combat the sense of isolation that has arisen during their caregiving experience, caregivers used video chats like FaceTime (Apple Inc) to connect with family members. Similarly, others used video platforms like Zoom for prayer groups, book clubs, music “jam” sessions, and church services, to maintain active in groups that they enjoyed prior to assuming the caregiving role. One participant praised virtual social-educational opportunities, remarking on a grant-funded opportunity where caregivers could take an online cooking class once a month,

*The girl would get on there once a month, and we would cook. Everybody... They would have us on camera and we would cook these nutrition meals. […] so, it was fun, because they would give us recipes and, and things like that. That was really good, because we can’t get out, you know?*

[P3; 67-year-old female participant].

Overall, technology can be leveraged to maintain a sense of normalcy for caregivers, providing them with the opportunity to connect with family and friends and to maintain personal interests, as they remain largely at home with the care recipient.

#### Subtheme 2: Promoting Wellness/Self-Care

Lastly, caregivers use technology as a form of self-care. Many remarked on the power of music as a tool to wind down both themselves and their care-recipient. One stated that her grandmother was very responsive to the calming effect of music during episodes of dementia-related agitation, noting:

*that’s an opportunity for sure to say* “*Alexa” - [when] my grandmother was having a meltdown, and that song started to play. I didn’t have to do a lot of effort to play it. Then usually she would stop and listen to that song*

[P17; 53-year-old female participant].

Similarly, another recalled that downloading favorite programs on their smart TV allowed both the caregiver and care recipient to enjoy time together despite limitations in mobility and being immunocompromised:

*So, I finally said, ‘You know what I’m going to download BritBox for you, and I’m going to download the PBS passport’ because we love watching the mysteries and stuff.*

[P8; 59-year-old male participant].

Others played digital games to distract themselves and relax, remarking that

*They [care recipients] are resting... it’s a time consumption, but it’s also, I feel like, at least I’m using my brain. So sometimes I feel like all I use is my brawn and not my brain.*

[P21; 79-year-old female participant].

Additional examples of technology-mediated self-care included guided meditation apps for relaxation and attending virtual music jam sessions and concerts, as described previously.

## Discussion

### Principal Findings

Caregivers of older homebound adults describe complicated perspectives on using technology in their caregiving. Nationally, most caregivers of older adults are younger than 64 years old [33]. That most caregivers in our study were older adults themselves presents a unique perspective on technology use in this population, and the tensions they encounter in incorporating technology into their caregiving activities. Our sample was interviewed as COVID-19 restrictions were easing, which reflects the unique experience of those who may have been technology-averse but were forced, due to circumstances created by the pandemic, to integrate modes of virtual interaction into their daily lives. Although most participants found that keeping health appointments with providers was beneficial, allowing them to learn new technologies such as Zoom and other virtual meeting dashboards, this did not translate into a general embrace of technology beyond instrumental activities of daily living such as banking, shopping, or medication management. While participants reported high levels of technology acceptance on the technology acceptance model questionnaire, the qualitative interviews revealed nuanced experiences characterized by frustration, uncertainty, and hesitation when engaging with specific technologies. This divergence may reflect differences between general attitudes toward technology and the practical realities of its use in everyday contexts [34,35]. Previous literature generally supports the notion that research participants are more forthcoming in reporting sensitive topics through anonymous survey questionnaires [36]. It is possible that the context of a group discussion normalized participants’ experiences [37] on the barriers they experience when using technology. While participants recognized the potential value of technology in improving communication, support, and some daily tasks, their narratives highlighted barriers to usability, confidence, technical difficulties, and needs for assistance. These findings suggest that technology acceptance should not be interpreted solely as a willingness to use technology but should also consider the contextual factors that influence sustained engagement. Our first theme aligns with previous work, indicating that physical impairments, in addition to dispositional and technical factors, may limit technology use [38,39]. Nonetheless, the subthemes represent a glaring gap for technologies that are designed expressly for caregivers—there are few that combine their shared concerns (safety, connection to others in their situation, local resources, and clinical expertise). That caregivers expressed a need for resources that combine multimodal types of support aligns with recent guidance to enhance reach and accessibility for technology among older adults [40,41]. Thus, co-designing technologies with caregivers leverages their unique experiential expertise, increasing the chances that digital solutions will not only address explicit caregiving needs but also the subtle, often invisible demands, workarounds, and emotional complexities that shape daily caregiving and are unlikely to be identified through researcher or clinician-driven design alone.

The second theme, Balancing the Scales, illustrated the sentiment echoed across participants that caregivers wear many hats in their role, often feeling like the odds are stacked against them. This theme addressed facilitators to technology use on a broad level, indicating that tailored education and training that meets caregivers where they are in their caregiving journey may address the barriers identified in Theme 1. Its related subthemes further highlighted drivers of technology use as issues of practicality. Using technology to assist with instrumental activities of daily living allows caregivers to simplify their daily routines as they carry a significant mental load in managing their homes and loved ones’ condition. The third subtheme, Promoting Sense of Security, centered around balancing caregivers’ sense of vigilance—the idea of always being present or “on duty”—with their needs for peace of mind. However, some participants expressed privacy invasion concerns regarding technologies used for surveillance of their loved ones. An important ethical concern for the use of technologies such as home monitoring devices for adults with dementia and cognitive impairment is the appropriate balance between autonomy and safety. A balance between protecting individuals from harm while preserving independence and dignity is essential and should be negotiated carefully [42].

Interestingly, participants did not describe the feeling of a 24/7 responsibility as a burden during their interviews, despite reporting moderate caregiver burden (ZBI-4 scores), but rather as an inherent feature of their situation. It may be that these caregivers have normalized their caregiving demands as an expression of commitment or responsibility due to their familial relation (spouse, adult child, and grandchild) to the care recipient.

The final theme, Preserving a Sense of Self, speaks to the idea that caregivers also have personally driven needs for technology use that may buffer the strains of caregiving. Although it was beyond the scope of this study to examine participants’ personal motivations for caregiving, it was evident that a sense of duty and obligation were underlying drivers and that the toll of the caregiving experience called for respite. The subtheme of Maintaining Social Connections and Promoting Wellness further enforces the importance of caregivers maintaining relationships and interests that are critical for their own identities and tempering the role loss experienced by caregivers that has been described by others [43,44]. That caregivers’ LSNS scores suggested that participants were experiencing social isolation aligns with this final theme, expressing needs for connection and support. Indeed, loneliness and isolation are pervasive among caregivers of older adults [45,46] and deleteriously affect their mental and physical health [47]. Left unaddressed, declines in caregiver health may negatively impact care recipients [48]. Interestingly, caregiver respondents rated their health as “good,” suggesting that caregivers may perceive themselves as physically healthy while nonetheless experiencing substantial psychosocial demands associated with caregiving. This distinction underscores the importance of assessing caregiver well-being across multiple domains, as self-rated health alone may not fully capture the emotional and social consequences of the caregiving role. Finally, despite the robust evidence-base of interventions to reduce loneliness and isolation for older adults, caregivers are less frequently the intended target population [49]. Our findings suggest that while caregivers value technology as a means of accessing information and support, digital tools are likely to be most effective when they foster meaningful human connection rather than replacing it.

### Limitations

There are several limitations to our study. First, our sampling frame draws from participants enrolled with one community service agency in Houston, which may not comprehensively represent the diversity of caregiving experiences or those in the general US population. Similarly, geographical differences in access to resources, internet connectivity, and linguistic preferences may have influenced adoption and use of technology among caregivers. As such, the linguistic ability within our research team and our partner organization was limited to English, so our study does not address important issues surrounding digital inclusivity and the application of these approaches across a variety of sociocultural situations.

That the majority of the sample comprised women reflects the larger picture of caregiving in the United States, where females disproportionately (55%) assume caregiving roles [50]. Further, variations in the treatment burden among caregivers according to the type, frequency, and intensity of care provided are difficult to discern, as we did not collect detailed information regarding the care recipients. Finally, since this study was conducted during the post-COVID context, it is difficult to disentangle the lasting consequences of the pandemic from those that reflect broader technological and societal trends in adopting technology in this population. Given the rapidly evolving technological landscape, participants’ experiences may reflect practices specific to the post-COVID period, which limits the transferability of the findings as digital tools and technology continue to change.

### Conclusions

Caregivers and their care recipients navigate chronic illness in-tandem, using available resources such as technology to alleviate the burdens of care. While technology has considerable potential to support caregiving, it is not a solution in and of itself. As reflected in participants’ narratives, technology can also impose additional burdens when it is difficult to use or fails to address their evolving needs. The present technological landscape requires that caregivers and older adults seek out and initiate the process of adopting new technology to integrate it into their lives; its potential is optimized when individuals are not only adequately trained but also fully aware of its capacities and limitations. Our study demonstrates that caregivers of homebound older adults adopt technology tools that reduce their concrete burdens such as time, travel, and monitoring. However, they underuse technologies that introduce financial, cognitive, or emotional costs. These dueling tensions should be recognized by researchers, clinicians, and technology developers as contextual determinants to using technology across the caregiving trajectory. Our findings highlight that targeted, multisectoral efforts toward digital inclusivity among vulnerable groups such as caregivers and their care recipients are sorely needed. Toward that end, it is imperative that health providers, social services clinicians, and tech developers are fully engaged with end users, such as caregivers, in co-design approaches that ensure not only the acceptability and usability of technology but that these technologies are ethically designed while considering caregivers’ everyday limitations, financial constraints, emotional workload, and digital confidence.

## Abbreviations

COREQ: Consolidated Criteria for Reporting Qualitative Research
HIPPA: Health Insurance Portability and Accountability Act
LSNS: Lubben Social Network Scale
ZBI-4: 4-item Zarit Burden Interview
